# Filamin A in focus: unravelling the multifaceted roles of filamin A in neurodevelopment and neurological disorders

**DOI:** 10.1093/brain/awaf180

**Published:** 2025-05-14

**Authors:** Longbo Zhang

**Affiliations:** Department of Neurosurgery, Xiangya Hospital, Central South University, Changsha 410008, China; National Clinical Research Center of Geriatric Disorders, Xiangya Hospital, Central South University, Changsha 410008, China

**Keywords:** filamin A (FLNA), neurodevelopment, neurodevelopmental disorders

## Abstract

Neurodevelopment is an intricate process encompassing the proliferation, differentiation, migration and maturation of neural cells. Disruptions in these tightly regulated events can lead to a variety of neurodevelopmental disorders. Filamin A (FLNA), a key actin-binding protein, plays a pivotal role in regulating neuronal migration, morphological development and synaptic connectivity by modulating actin cytoskeletal dynamics and interacting with various signalling pathways. *FLNA* mutations are implicated in several neurodevelopmental disorders, such as periventricular nodular heterotopia (PVNH), leading to neurological symptoms such as epilepsy, intellectual disability and cognitive impairments.

In this review, we delve into FLNA’s multifaceted role in neurodevelopment, with a particular focus on its contributions to neuronal migration, dendritic and axonal growth and mechanotransduction. Additionally, we examine how FLNA dysregulation leads to neurodevelopmental abnormalities, providing insights into its potential as a therapeutic target. By elucidating the molecular mechanisms through which FLNA governs neurodevelopment, we aim to advance our understanding of its crucial role in both brain formation and disease pathogenesis.

## Teaser

Mutations in the gene encoding the cytoskeletal protein *FLNA* are linked to disorders such as epilepsy and autism. Longbo Zhang explores the role of FLNA in brain development, focusing on neuronal migration and dendritic and axonal growth and examines how dysregulation of this protein contributes to neurodevelopmental abnormalities.

## Introduction

Neurodevelopment is a highly orchestrated process that begins early in embryogenesis and continues through adolescence, encompassing the formation, maturation and specialization of the nervous system. This intricate process involves a series of tightly regulated events, including cell proliferation, differentiation, migration, synaptogenesis, myelination and neuroapoptosis.^1^ The proper execution of these events is critical for the formation of a functional brain, and disruptions at any stage can lead to a variety of neurodevelopmental disorders.^2^

Filamin A (FLNA), a critical component of the cytoskeletal system, is a large actin-binding protein that plays a dual role in cellular architecture and signalling. It organizes actin filaments into orthogonal, 3D networks, providing mechanical stability and enabling cells to maintain their shape and resist mechanical stress. FLNA also facilitates the dynamic remodelling of the actin cytoskeleton, a process essential for cellular activities such as migration, adhesion and cytokinesis. Furthermore, FLNA serves as a scaffold for numerous signalling molecules, thereby integrating cytoskeletal dynamics with intracellular signalling pathways and mediating complex cellular responses.^3,4^ During brain development, FLNA is integral to maintaining cell shape, transmitting mechanical signals and orchestrating the migration and positioning of neurons.^5^ In addition, FLNA regulates neuronal morphological development by influencing dendritic and dendritic spine formation through its impact on actin networks. FLNA also governs axonal growth and guidance by modulating actin dynamics in growth cones, ensuring accurate axon extension and pathfinding.^6-8^ Beyond structural support, FLNA is deeply involved in the signalling pathways that guide neurons through the complex environment of the developing brain. By binding to and modulating a diverse array of proteins, FLNA influences essential cellular processes such as signal transduction and mechanosensing.^9,10^ These processes are crucial for ensuring that neurons reach their correct locations and undergo proper morphological development within the brain, thereby establishing appropriate connections with other neurons.

Mutations in the *FLNA* gene, which encodes the FLNA protein, are linked to various neurodevelopmental disorders. The most prominent of these is periventricular nodular heterotopia (PVNH), characterized by the abnormal accumulation of neurons that fail to migrate correctly during brain development.^11,12^ Disruptions of FLNA are also associated with other conditions such as focal cortical malformations (FCMs) and autism spectrum disorder (ASD), presenting a diverse range of neurological symptoms, including epilepsy, intellectual disability and cognitive impairments.^7,8,13-15^ Therefore, elucidating the molecular mechanisms through which FLNA influences neurodevelopment is essential for advancing both basic neuroscience and clinical research.

In this review, we explore the multifaceted functions of FLNA in neurodevelopment, elucidating its contributions to neuronal migration, morphology and signal transduction. We also examine mechanisms of FLNA regulation and its implications for neurodevelopmental and neurodegenerative disorders. By evaluating the current research on FLNA, we aim to enhance our understanding of its pivotal role in brain development and pathology, ultimately informing innovative therapeutic strategies.

## Molecular regulation in neurodevelopment

Neurodevelopment is a highly complex and coordinated process involving the maturation and interaction of various neural cell types, which shape the formation and function of normal neural circuits and networks. It begins with neurulation, where the neural tube forms the foundation of the brain and spinal cord. From there, neurogenesis generates billions of neurons that embark on an adventurous migration to their designated locations, followed by differentiation into specialized roles. Synaptogenesis weaves intricate networks of connections, while myelination enhances communication speed by insulating nerve fibres. Through pruning, the brain refines these networks, trimming away excess connections to ensure optimal performance.^2,16^ This entire process unfolds during critical periods, where the precise expression and function of key proteins and signalling pathways regulate the cellular and molecular mechanisms essential for neural development, including the positioning, differentiation and connectivity of neurons and glial cells.^17,18^ For instance, the PI3K/AKT/mTOR pathway, representing a pivotal regulator of the homeostatic balance of protein synthesis during neurodevelopment, governs critical processes such as neuronal progenitor proliferation, differentiation and neurite outgrowth and elongation.^19^ Molecules regulating cytoskeletal dynamics, including RhoA and RAC1, play crucial roles in facilitating neuronal movement, growth and structural adaptations.^20^ FLNA could modulate actin organization, influencing neuronal migration, neurite branching and axon guidance, all of which are fundamental for neurodevelopment.^6-8^ These processes are intricately coordinated through the regulation of mRNA translation and cell cycle progression and exit.^19,21^ In this context, key regulators of the cell cycle, including cyclin B1 and Cdk1, play essential roles in ensuring the timely division of neural progenitor cells, further contributing to the precise orchestration of neurodevelopmental processes.^22^ By regulating the transcript levels of developmental genes in the brain, transcriptional regulators such as MECP2 and CHD8 control the maturation of cortical inhibitory and excitatory connections during development, as well as the regulatory networks that drive neuronal specification and activity-dependent responses.^23,24^ FLNA also participates in transcription and translation processes, potentially influencing gene expression critical for neuronal differentiation and plasticity.^25^ Given the multiple targets of each of these proteins, dysregulation of any one of them can result in pleiotropic effects.^2,26,27^ Additionally, during neurodevelopment, two primary categories of synaptic proteins are critical for the activity-dependent establishment of neuronal circuits: cell-adhesion molecules (CAMs), which orchestrate the bidirectional organization of pre- and postsynaptic compartments through trans-cellular signalling,^28^ and scaffolding and synaptic signalling proteins, such as neurexins (NRXN), neuroligins (NLGN) and SHANKs, which are situated at the postsynaptic density and form extensive molecular networks involving receptors and actin-associated proteins.^29^ FLNA, as a cytoskeletal scaffold, is instrumental in anchoring synaptic proteins to the actin network, thereby facilitating synaptic stabilization and function. FLNA mutations have been linked to synaptic deficits and aberrant excitatory-inhibitory balance.^30-32^ Furthermore, proteins such as Big2-Arf coordinate vesicle trafficking and membrane dynamics, which are essential for the proper localization of proteins and receptors necessary for synaptic function.^5^ Therefore, proper regulation of these proteins is essential for establishing functional neural networks. Disruptions in their expression or function can impair neurodevelopment, contributing to various neurological and psychiatric disorders such as ASD and intellectual disabilities.^2^ Understanding the roles of these key proteins in neuronal and glial development is crucial for deciphering the mechanisms underlying normal brain function and the pathogenesis of neurodevelopmental diseases.

## FLNA exhibits versatility in cellular structure and signalling

**Figure 1:**
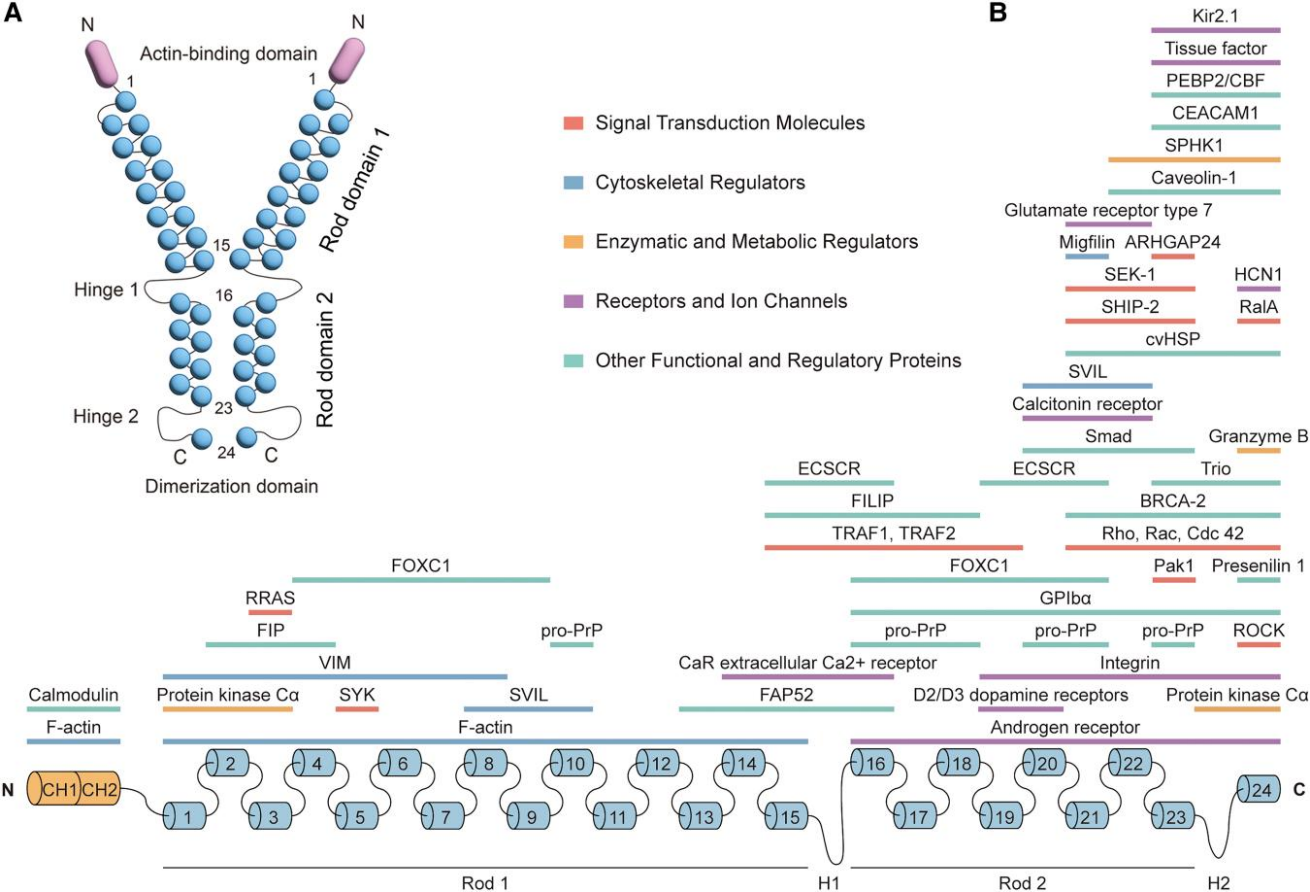
**Schematic representation of filamin a and binding sites of interaction partners.** (**A**) The N-terminal actin-binding domain is succeeded by 24 immunoglobulin-like (Ig) repeats. These repeats are divided into two rod domains by two hinge regions: Rod Domain 1 consists of repeats 1–15, while Rod Domain 2 encompasses repeats 16–24. The C-terminal 24th repeat functions as the dimerization domain (modified from Razinia *et al*.^45^). (**B**) The classification of interaction partners and their respective ligand-binding sites (updated from Feng *et al*.^9^ and Zhou *et al*.^43^).

FLNA, a 280-kDa actin-binding protein, plays a crucial role in crosslinking actin filaments into orthogonal networks within the cytoplasm. It is instrumental in anchoring membrane proteins to the actin cytoskeleton and is recognized as a multifunctional, large dimeric protein that is essential for various cellular processes.^3^ Structurally, FLNA comprises two primary structural domains: an N-terminal actin-binding domain (ABD) and a C-terminal region consisting of 24 immunoglobulin-like (Ig) repeats organized into rod-like segments. The ABD, which includes two calponin homology domains, is critical for high-affinity interaction with F-actin. Rod segment 1, with its extended, flexible structure, stabilizes this interaction and imparts rigidity to the actin network. In contrast, rod segment 2 features a more compact, globular configuration that facilitates dimerization and orthogonal actin network formation^9,33^ (Fig. 1A). FLNA’s capacity to cross-link actin filaments orthogonally, coupled with its role in mediating interactions with other cellular components, ensures structural integrity, supports cellular shape changes and maintains mechanical stability under stress.^34^ In addition, FLNA can link actin dynamics with various signalling pathways and interact with a wide range of cellular proteins and molecules with diverse functions, including membrane receptors, enzymes, channels, signalling intermediates and transcription factors, and it modulates the functional activities of these binding partners. These interactions play an indispensable role in regulating cell adhesion, migration and mechanosensing by mediating the assembly of protein complexes involved in these processes, suggesting that FLNA functions as an unusually versatile signalling scaffold^4,9,33,35,36^ (Fig. 1B and Supplementary Table 1). Beyond its structural and signalling roles, FLNA also influences gene expression by translocating to the nucleus and interacting with transcription factors, thereby affecting rRNA expression patterns crucial for cell differentiation and stress responses.^25,37^ The functional versatility of FLNA is further enhanced by its range of post-translational modifications (PTMs) and alternative splicing. Alternative splicing of FLNA generates distinct isoforms with specialized roles in cytoskeletal organization, cell migration and signalling, which are developmentally regulated and critical for neuronal and non-neuronal processes. Together with PTMs, such as phosphorylation, irreversible cleavage, ubiquitin-mediated degradation, hydroxylation and *O*-GlcNAcylation, splicing enables FLNA to coordinate cellular architecture and modulate its extensive protein-protein interaction network, central to its role as a cellular signalling hub. Dysregulation of these mechanisms, including emerging PTMs like tyrosination, carbonylation and acetylation, contributes to various diseases, including cancer, cardiovascular disorders and neurological conditions.^38-41^

## FLNA in neurodevelopment

In neurodevelopment, FLNA is pivotal in orchestrating key cellular processes vital for brain formation and organization. It plays a critical role in regulating neuronal maturation and migration, ensuring that neurons reach their precise locations within the developing brain,^5,22,42,43^ which is essential for establishing the intricate architecture necessary for functional neural circuits. FLNA is also integral to axonal and dendritic growth and guidance, facilitating the formation of synaptic connections that support neural network function.^6-8^ FLNA provides cellular stability by reinforcing the plasma membrane and cytoskeleton under mechanical stress. It localizes to the cell cortex, enhances adhesion site stability and increases in response to applied force. FLNA also plays a role in resisting fluid shear stress and maintains membrane integrity through its interaction with GPIbα.^44,45^

### FLNA regulates neuronal progenitor proliferation and differentiation

During cortical development, neural progenitors migrate to the subventricular zone, where they generate neurons that move along radial glial fibres to form cortical layers. As development progresses, progenitors adopt symmetric divisions to produce terminal neurons and astrocytes.^45^ FLNA is plays a significant role in the regulation of neural progenitor cell proliferation. During embryonic development, FLNA disruption reduces brain size and neural progenitor numbers by extending the cell cycle, not through increased cell death or premature differentiation. Specifically, FLNA loss impairs cyclin B1 degradation, causing delays in mitosis due to increased Cdk1 phosphorylation and reduced anaphase-promoting complex activity.^22^ Additionally, FLNA influences progenitor specification by promoting vertical cleavage planes during cell division, which increases symmetric divisions and decreases the expression of early neuronal markers. FLNA interacts with RhoA, and its loss results in reduced RhoA activation and altered Aurora kinase B localization. This disruption leads to delayed neurogenesis, prolonged progenitor proliferation, and affects cell fate and cytokinesis, thereby modulating neural progenitor differentiation.^20^

### FLNA in neuronal migration

The orchestrated migration of neurons is critical for the proper layering of the cerebral cortex and the accurate formation of brain regions, both of which are essential for establishing the brain's functional architecture. By coordinating the polymerization and crosslinking of actin filaments, FLNA facilitates the formation of cellular protrusions, such as lamellipodia and filopodia, which are crucial for the directed movement of neurons.^5,46^ Additionally, the 3D network formed by FLNA-crosslinked actin filaments provides the mechanical strength necessary to ensure that migrating neurons can precisely traverse radial glial fibres to reach cortical layers.^47^ Beyond guiding individual neuronal movements, FLNA’s regulation of the actin cytoskeleton ensures that neurons possess the structural flexibility required for migration.^3,4,9^ This flexibility is especially critical as neurons maneuver through the dense extracellular matrix and interact with other migrating cells. Furthermore, FLNA possibly contributes to neuronal migration by coordinating interactions between actin and vesicle trafficking (via Big2-Arf), facilitating the assembly and disassembly of membrane protein complexes essential for neuronal migration and neuroependymal integrity. Disruption of FLNA function can lead to abnormal neuronal positioning, resulting in cortical malformations and associated neurodevelopmental disorders, highlighting the indispensable role of FLNA in brain development.^5^

### FLNA in neuronal morphology

FLNA plays a pivotal role in axonal growth and guidance, processes essential for the formation of functional neural circuits. Neurite extension and axon growth depend on the coordinated actions of the actin and microtubule cytoskeletal networks. Neurites and axons contain bundled microtubules, surrounded by an actin-rich network in the growth cone, a dynamic structure at the axon’s leading edge. Actin dynamics enable microtubules to advance toward the growth cone periphery, with retrograde actin flow facilitating microtubule extension and neurite formation from spherical neurons.^48-50^ By regulating these actin dynamics, FLNA orchestrates cytoskeletal remodelling, driving axonal extension and directional movement. This ensures axons respond to guidance cues, reach their target destinations and establish proper synaptic connections.^6^ In addition to its role in axonal guidance, FLNA is critically involved in dendritic growth and development, processes that shape the receptive fields and influence synaptic integration.^8^ FLNA’s regulation of actin dynamics extends to dendrites, impacting dendritic complexity and length, the factors crucial for the formation of functional synapses. By fine-tuning dendritic morphology, FLNA contribute to the proper organization of dendritic arbors, which is necessary for the integration of synaptic inputs and the establishment of effective neural networks.^8^ Notably, similar to its role in regulating migration,^51-53^ an optimal level of FLNA is required to balance dendritic growth and branching, underscoring its importance in maintaining the structural and functional integrity of neuronal circuits.^8,54^ Interestingly, FLNA’s regulation of dendritic morphology might be indirect, potentially mediated through the modulation of other molecular proteins, as studies have shown that FLNA lacking the ABD can still regulate dendritic development.^8^ Furthermore, FLNA stabilization promotes dendritic spine maturation and remodelling, critical for synapse formation and maintenance.^7,55,56^ FLNA also regulates dendritogenesis and spinogenesis by modulating RAC1 and cofilin through its interaction with Rho-GTPase activating protein 24 (ARHGAP24), essential for maintaining excitatory-inhibitory balance in neural circuits.^42^

### FLNA in mechanotransduction and signalling

FLNA is involved in the process by which cells convert mechanical stress into biochemical signals and plays a central role in the neuronal response to physical forces within the brain’s microenvironment.^3,57^ FLNA integrates mechanical signals with cytoskeletal dynamics, enabling neurons to adapt their morphology, motility and behaviour in response to environmental changes such as mechanical forces, extracellular matrix stiffness and cell-cell interactions.^58-60^ This adaptability is crucial for neural network development and the brain's plasticity in response to injury or changing functional demands.^61^ Serving as an signalling interaction platform, FLNA’s role in mechanotransduction also extends to the regulation of gene expression and cellular signalling pathways,^45^ allowing FLNA to not only contribute to neuronal structural development but also contribute processes such as cell differentiation, survival and synaptic plasticity.^7,22^ For example, FLNA interacts with key signalling molecules in pathways such as PI3K/Akt and RhoA/ROCK, guiding the differentiation of neural progenitor cells into specific neuron types with distinct functions,^20^ ensuring that neurons acquire the correct identities and functional properties necessary for their roles within the neural network. Additionally, FLNA is involved in coordinating cytoskeletal dynamics and signalling pathways related to synaptic signalling,^42,62^ thereby regulating synaptic plasticity and connectivity, processes that are essential for learning and memory. Dysregulation of FLNA function can impair mechanotransduction and disrupt the interaction between intracellular and extracellular signalling pathways, potentially leading to developmental abnormalities and neurological disorders.

Overall, FLNA’s intricate structure and diverse functions underscore its importance in preserving cellular integrity and orchestrating neurodevelopmental processes. By linking actin filaments to membrane receptors and interacting with a broad signalling proteins, FLNA plays a crucial role in neuronal migration, axonal guidance and the formation of functional neural networks. This coordination is vital for establishing a well-organized and interconnected brain, which is essential for normal cognitive and motor functions. Disruptions in FLNA function can impair these processes, leading to developmental and neurological disorders.

## FLNA and associated neurodevelopmental disorders

Disruptions in FLNA function have been implicated in a broad range of neurodevelopmental disorders leading to aberrant neuronal positioning, impaired synaptic formation and connectivity deficits, which collectively contribute to the pathophysiology of various neurodevelopmental conditions such as PVNH, epilepsy and other related cognitive disorders.^63^

### FLNA mutations and periventricular nodular heterotopia

**Figure 2:**
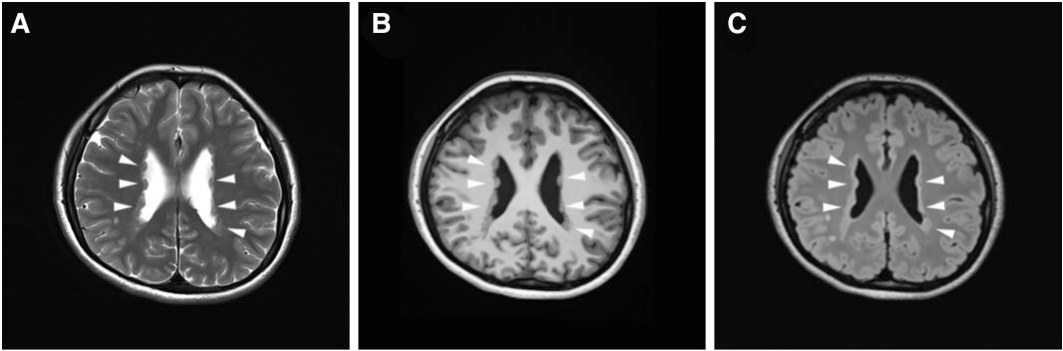
**Imaging features of periventricular nodular heterotopia (PVNH).** PVNH patient: MRI T2-weighted (**A**), multiplanar reformation (MPR; **B**) and T2-weighted dark fluid (**C**) images reveal abnormal nodules of grey matter (PVNH) adjacent to the bilateral lateral ventricles (indicated by arrows).

PVNH is an X-linked neurodevelopmental disorder characterized by the presence of neuron clusters abnormally positioned near the ventricles rather than within the cortical layers where they would typically reside (Fig. 2). This aberrant neuronal positioning results from disruptions in the normal migration process during brain development, specifically from the ventricular zone to the cortex.^64,65^ Mutations in the *FLNA* gene are well-established causes of PVNH, typically resulting from the loss-of-function of one *FLNA* allele, leading to abnormal mRNA splicing or early truncation of the FLNA protein.^66,67^ For example, specific truncating mutations in *FLNA* at C-terminal region disrupt key interactions with membrane receptors and intracellular signalling molecules, including integrins and other ligands essential for cell adhesion and migration. These mutations impair the proper migration of neurons along radial glial fibres, causing the formation of heterotopic nodules, where neurons fail to reach their intended cortical destinations.^47^ Additionally, FLNA may function as a scaffold in cellular signalling, with PVNH potentially arising from disrupted programmed cell death or continued neuroblast proliferation. This is evidenced by a specific *FLNA* mutation that truncates the protein at amino acid 2305, disrupting key interactions with membrane receptors and signalling molecules.^9^ PVNH is frequently associated with a range of neurological and cognitive symptoms, including epilepsy, intellectual disability and motor impairments. Epilepsy, the major clinical syndrome observed in individuals with PVNH, is often resistant to conventional treatments, posing a significant challenge and negatively impacting quality of life.^11^ The severity of intellectual disability and motor impairments in PVNH can vary widely, depending on the extent and distribution of neuronal misplacement. The phenotypic variability observed in PVNH highlights the complexity of the disorder and underscores the crucial role that *FLNA* mutations play in neural development.^68^ Ongoing research seeks to unravel the mechanisms by which these mutations lead to such diverse clinical outcomes, with the aim of developing targeted therapies that address the underlying causes of the disorder and improve patient outcomes. The study of PVNH not only sheds light on the specific consequences of *FLNA* mutations but also contributes to a broader understanding of the fundamental processes governing neuronal migration and cortical development.

### FLNA abnormalities associated with focal cortical malformations

Recent findings suggest a potential association between FLNA abnormalities and FCMs, such as focal cortical dysplasia (FCD) and tuberous sclerosis complex (TSC). FCD is a neurodevelopmental disorder characterized by abnormal development and organization of cortical neurons, often resulting in epilepsy with seizures originating from the affected cortical areas. Key features of FCD include disorganized neuronal clusters and aberrant cortical layers, which disrupt normal brain function. A missense variant of *FLNA* (c.1604A>G: p.D535G) has been identified in FCDII patients with right frontal lobe epilepsy.^69^ Furthermore, studies involving larger patient cohorts and murine models have demonstrated elevated FLNA transcription levels.^8,13^ These FLNA abnormalities lead to disrupted dendritic development including excessive dendritic growth and increased complexity and neuronal misplacement, causing significant pathological changes in neuronal morphology and excitability. Such disruptions affect neural circuitry and increase susceptibility to seizures. However, a direct correlation between FLNA dysfunction and FCD remains to be fully established. FLNA’s regulation of dendritic growth and branching may involve critical interactions with actin-binding and cytoskeletal-associated proteins, such as Rho GTPases (e.g. Rac1 and Cdc42), which coordinate the remodelling of actin and microtubules. Disruptions in these pathways can lead to aberrant dendritic morphology.^3,70,71^ Of note, FLNA’s regulation of dendritic growth exhibits a bell-shaped pattern, indicating that proper FLNA expression is crucial for dendritic development during brain maturation.^8^ Additionally, FLNA might regulate dendritic morphology through its scaffolding role, indirectly modulating proteins involved in synaptic and cytoskeletal signalling. Recent evidence suggests FLNA interacts with microtubule-associated proteins and signalling complexes, further influencing dendritic architecture and dynamics.^30,72^ Research indicates that FLNA can still affect dendritic development even in the absence of ABD domain.^8^ Our previous study has demonstrated that targeting FLNA as a promising therapeutic strategy to address these neural abnormalities and significantly reduce the frequency of seizures.^13^ However, this concept remains to be validated in clinical settings. It is important to note that the mTOR signalling pathway, as a critical regulator of neurodevelopmental processes including neuronal progenitor proliferation, differentiation and dendritic growth, is well-studied in the context of FCD and TSC. Recent studies suggest that FLNA may function as a scaffolding protein that modulates actin and microtubule dynamics involved in these disease processes, potentially linking its role to the mTOR pathway, albeit in a MEK-ERK1/2-dependent and mTOR-independent manner.^8,21,73^

### FLNA dysfunction linked to autism spectrum disorder

ASD encompasses a wide range of cognitive and behavioural abnormalities, with various genetic factors implicated in their development. Neurodevelopmental disorders significantly contribute to ASD through altered neurogenesis, abnormal neuronal migration and impaired neuronal connectivity.^15,74^  ^,75^ Neuronal migration disorders, for example, are a general feature of ASD. MRI studies have frequently identified various brain malformations associated with neuronal migration disorders in individuals with ASD, including: altered volumes in the cerebellum, frontal lobe and limbic system; enlarged ventricles; reduced cortex thickness; heterotopias; and abnormal cortical gyrification.^76,77^ More direct evidence of migration deficits includes MRI-detectable structural abnormalities such as lissencephaly and heterotopias, which are linked to abnormal radial migration in the developing brain. Reports frequently associate ASD with pachygyria, dysplasia and heterotopia resulting from PVNH and FCMs, conditions that are linked to disruptions in FLNA protein.^32,78,79^ Additionally, FLNA’s involvement in the structure and function of dendritic spines and neuronal connectivity is increasingly recognized as a factor contributing to the diverse behavioural and cognitive symptoms observed in ASD.^80^ For instance, communication impairments in ASD are often linked to early defects in vocalizations.^81,82^ Recent studies using an mTORopathy model have shown altered vocalizations and dendrite overgrowth, alongside increased spine density and length, indicating excessive excitatory activity. While reducing FLNA levels restored both spine properties and vocalizations, this indicates a potential regulatory role of FLNA in dendritic and synaptic changes^7^; however, direct pathogenic variants of FLNA causative for ASD have not conclusively been identified.

Together, FLNA disruptions are associated with a diverse array of disorders that affect neurological development. The wide range of symptoms, including dysmorphism, seizures, developmental delays and cognitive impairments, underscores FLNA’s pivotal role in orchestrating complex developmental processes. Notably, FLNA pathogenic variants, while extensively studied for their role in neurodevelopmental disorders, are also implicated in a range of neurodegenerative diseases, including Alzheimer’s disease and tauopathies,^72,83-85^ as well as various non-neurological pathologies. Among the most documented are cardiovascular abnormalities, such as patent ductus arteriosus, alongside gastrointestinal complications like congenital intestinal pseudo-obstruction and organ malrotation.^86-90^ Skeletal and connective tissue disorders, including Ehlers-Danlos-like syndromes and scoliosis, are also frequently observed in individuals with FLNA mutations.^89,90^ Additionally, respiratory issues such as pulmonary hypertension and bronchomalacia further broaden the spectrum of associated conditions.^91-93^ Addressing these diverse manifestations is vital for comprehensive patient care and highlights the need for further research into the molecular mechanisms underlying FLNA-related pathologies.

## Conclusion

Molecular regulation during neurodevelopment is essential for establishing normal brain architecture and function. FLNA plays a critical role in this process by coordinating key cellular mechanisms involved in neuronal migration, axonal growth, dendritic development and synaptic connectivity. FLNA also acts as a versatile scaffold, linking actin filaments to membrane receptors and interacting with a broad range of signalling molecules to regulate cell adhesion, migration and mechanotransduction. Disruptions in FLNA function can lead to abnormal neuronal positioning, impaired synaptic formation and altered connectivity, resulting in a spectrum of neurodevelopmental disorders, including PVNH, FCMs and ASD. A comprehensive understanding of FLNA’s multifaceted roles in neural development and disease can elucidate its crucial function in preserving the brain's structural and functional integrity. This knowledge is essential for unravelling the pathogenesis of diverse neurological disorders and for devising precise therapeutic interventions.

## Supplementary Material

awaf180_Supplementary_Data

## References

[1] Stiles J, Jernigan TL. The basics of brain development. Neuropsychol Rev. 2010;20:327–348. 21042938 10.1007/s11065-010-9148-4 PMC2989000

[2] Parenti I, Rabaneda LG, Schoen H, Novarino G. Neurodevelopmental disorders: From genetics to functional pathways. Trends Neurosci. 2020;43:608–621. 32507511 10.1016/j.tins.2020.05.004

[3] Nakamura F, Stossel TP, Hartwig JH. The filamins: Organizers of cell structure and function. Cell Adh Migr. 2011;5:160–169. 21169733 10.4161/cam.5.2.14401 PMC3084982

[4] Popowicz GM, Schleicher M, Noegel AA, Holak TA. Filamins: Promiscuous organizers of the cytoskeleton. Trends Biochem Sci. 2006;31:411–419. 16781869 10.1016/j.tibs.2006.05.006

[5] Sarkisian MR, Bartley CM, Rakic P. Trouble making the first move: Interpreting arrested neuronal migration in the cerebral cortex. Trends Neurosci. 2008;31:54–61. 18201775 10.1016/j.tins.2007.11.009

[6] Cho Y, Park D, Cavalli V. Filamin A is required in injured axons for HDAC5 activity and axon regeneration. J Biol Chem. 2015;290:22759–22770. 26157139 10.1074/jbc.M115.638445 PMC4566247

[7] Binder MS, Escobar I, Xu Y, Sokolov AM, Zhang L, Bordey A. Reducing filamin A restores cortical synaptic connectivity and early social communication following cellular mosaicism in ASD pathways. J Neurosci. 2024;44:e1245232024. 39164108 10.1523/JNEUROSCI.1245-23.2024 PMC11426378

[8] Zhang L, Bartley CM, Gong X, et al MEK-ERK1/2-dependent FLNA overexpression promotes abnormal dendritic patterning in tuberous sclerosis independent of mTOR. Neuron. 2014;84:78–91. 25277454 10.1016/j.neuron.2014.09.009 PMC4185153

[9] Feng Y, Walsh CA. The many faces of filamin: A versatile molecular scaffold for cell motility and signalling. Nat Cell Biol. 2004;6:1034–1038. 15516996 10.1038/ncb1104-1034

[10] Ehrlicher AJ, Nakamura F, Hartwig JH, Weitz DA, Stossel TP. Mechanical strain in actin networks regulates FilGAP and integrin binding to filamin A. Nature. 2011;478:260–263. 21926999 10.1038/nature10430 PMC3204864

[11] Battaglia G, Chiapparini L, Franceschetti S, et al Periventricular nodular heterotopia: Classification, epileptic history, and genesis of epileptic discharges. Epilepsia. 2006;47:86–97. 16417536 10.1111/j.1528-1167.2006.00374.x

[12] Sheen VL, Dixon PH, Fox JW, et al Mutations in the X-linked filamin 1 gene cause periventricular nodular heterotopia in males as well as in females. Hum Mol Genet. 2001;10:1775–1783. 11532987 10.1093/hmg/10.17.1775

[13] Zhang L, Huang T, Teaw S, et al Filamin A inhibition reduces seizure activity in a mouse model of focal cortical malformations. Sci Transl Med. 2020;12:eaay0289. 32075941 10.1126/scitranslmed.aay0289 PMC12290962

[14] Zhang L, Huang T, Bordey A. Tsc1 haploinsufficiency is sufficient to increase dendritic patterning and Filamin A levels. Neurosci Lett. 2016;629:15–18. 27345385 10.1016/j.neulet.2016.06.037 PMC4983256

[15] Wegiel J, Kuchna I, Nowicki K, et al The neuropathology of autism: Defects of neurogenesis and neuronal migration, and dysplastic changes. Acta Neuropathol. 2010;119:755–770. 20198484 10.1007/s00401-010-0655-4 PMC2869041

[16] Zhou Y, Song H, Ming GL. Genetics of human brain development. Nat Rev Genet. 2024;25:26–45. 37507490 10.1038/s41576-023-00626-5 PMC10926850

[17] Tau GZ, Peterson BS. Normal development of brain circuits. Neuropsychopharmacology. 2010;35:147–168. 19794405 10.1038/npp.2009.115 PMC3055433

[18] Jiang XN, Nardelli J. Cellular and molecular introduction to brain development. Neurobiol Dis. 2016;92:3–17. 26184894 10.1016/j.nbd.2015.07.007 PMC4720585

[19] Lipton JO, Sahin M. The neurology of mTOR. Neuron. 2014;84:275–291. 25374355 10.1016/j.neuron.2014.09.034 PMC4223653

[20] Lian G, Wong T, Lu J, Hu J, Zhang J, Sheen V. Cytoskeletal associated filamin A and RhoA affect neural progenitor specification during mitosis. Cereb Cortex. 2019;29:1280–1290. 29462287 10.1093/cercor/bhy033 PMC6499011

[21] Crino PB . The mTOR signalling cascade: Paving new roads to cure neurological disease. Nat Rev Neurol. 2016;12:379–392. 27340022 10.1038/nrneurol.2016.81

[22] Lian GW, Lu J, Hu JJ, et al Filamin A regulates neural progenitor proliferation and cortical size through wee1-dependent Cdk1 phosphorylation. J Neuroscience. 2012;32:7672–7684. 22649246 10.1523/JNEUROSCI.0894-12.2012 PMC3368379

[23] Suetterlin P, Hurley S, Mohan C, et al Altered neocortical gene expression, brain overgrowth and functional over-connectivity in haploinsufficient mice. Cereb Cortex. 2018;28:2192–2206. 29668850 10.1093/cercor/bhy058 PMC6018918

[24] Witteveen JS, Willemsen MH, Dombroski TC, et al Haploinsufficiency of MeCP2-interacting transcriptional co-repressor SIN3A causes mild intellectual disability by affecting the development of cortical integrity. Nat Genet. 2016;48:877–887. 27399968 10.1038/ng.3619

[25] Deng W, Lopez-Camacho C, Tang JY, et al Cytoskeletal protein filamin A is a nucleolar protein that suppresses ribosomal RNA gene transcription. Proc Natl Acad Sci U S A. 2012;109:1524–1529. 22307607 10.1073/pnas.1107879109 PMC3277164

[26] Moreau CA, Kumar K, Harvey A, et al Brain functional connectivity mirrors genetic pleiotropy in psychiatric conditions. Brain. 2023;146:1686–1696. 36059063 10.1093/brain/awac315 PMC10319760

[27] Woodward DJ, Thorp JG, Middeldorp CM, Akosile W, Derks EM, Gerring ZF. Leveraging pleiotropy for the improved treatment of psychiatric disorders. Mol Psychiatry. 2025;30:705–721. 39390223 10.1038/s41380-024-02771-7 PMC11746150

[28] Südhof TC . Towards an understanding of synapse formation. Neuron. 2018;100:276–293. 30359597 10.1016/j.neuron.2018.09.040 PMC6226307

[29] Bourgeron T . From the genetic architecture to synaptic plasticity in autism spectrum disorder. Nat Rev Neurosci. 2015;16:551–563. 26289574 10.1038/nrn3992

[30] Lee G, Schwarz TL. Filamin, a synaptic organizer in Drosophila, determines glutamate receptor composition and membrane growth. Elife. 2016;5:e19991. 27914199 10.7554/eLife.19991 PMC5173320

[31] Jobin ML, Siddig S, Koszegi Z, et al Filamin A organizes gamma-aminobutyric acid type B receptors at the plasma membrane. Nat Commun. 2023;14:34. 36596803 10.1038/s41467-022-35708-1 PMC9810740

[32] Sakai Y, Shaw CA, Dawson BC, et al Protein interactome reveals converging molecular pathways among autism disorders. Sci Transl Med. 2011;3:86ra49. 10.1126/scitranslmed.3002166 PMC3169432 21653829

[33] Gardel ML, Nakamura F, Hartwig JH, Crocker JC, Stossel TP, Weitz DA. Prestressed F-actin networks cross-linked by hinged filamins replicate mechanical properties of cells. Proc Natl Acad Sci U S A. 2006;103:1762–1767. 16446458 10.1073/pnas.0504777103 PMC1413620

[34] Nakamura F, Song M, Hartwig JH, Stossel TP. Documentation and localization of force-mediated filamin A domain perturbations in moving cells. Nat Commun. 2014;5:4656. 25120197 10.1038/ncomms5656 PMC4139033

[35] Nakamura F, Pudas R, Heikkinen O, et al The structure of the GPIb-filamin A complex. Blood. 2006;107:1925–1932. 16293600 10.1182/blood-2005-10-3964 PMC1895705

[36] Nakamura F, Hartwig JH, Stossel TP, Szymanski PT. Ca2 ^+^ and calmodulin regulate the binding of filamin A to actin filaments. J Biol Chem. 2005;280:32426–32433. 16030015 10.1074/jbc.M502203200

[37] Nguyen LXT, Chan SM, Ngo TD, et al Interaction of TIF-90 and filamin A in the regulation of rRNA synthesis in leukemic cells. Blood. 2014;124:579–589. 24850755 10.1182/blood-2013-12-544726

[38] Shead KD, Salyahetdinova V, Baillie GS. Charting the importance of filamin A posttranslational modifications. Biochem J. 2024;481:865–881. 38958472 10.1042/BCJ20240121 PMC11346442

[39] Robertson SP . Filamin A: Phenotypic diversity. Curr Opin Genet Dev. 2005;15:301–307. 15917206 10.1016/j.gde.2005.04.001

[40] Zhang X, Chen MH, Wu X, et al Cell-type-specific alternative splicing governs cell fate in the developing cerebral Cortex. Cell. 2016;166:1147–1162.e15. 27565344 10.1016/j.cell.2016.07.025 PMC5248659

[41] Oda H, Sato T, Kunishima S, et al Exon skipping causes atypical phenotypes associated with a loss-of-function mutation in FLNA by restoring its protein function. Eur J Hum Genet. 2016;24:408–414. 26059841 10.1038/ejhg.2015.119 PMC4755370

[42] Falace A, Corbieres L, Palminha C, et al FLNA regulates neuronal maturation by modulating RAC1-Cofilin activity in the developing cortex. Neurobiol Dis. 2024;198:106558. 38852754 10.1016/j.nbd.2024.106558

[43] Zhou AX, Hartwig JH, Akyurek LM. Filamins in cell signaling, transcription and organ development. Trends Cell Biol. 2010;20:113–123. 20061151 10.1016/j.tcb.2009.12.001

[44] Shifrin Y, Arora PD, Ohta Y, Calderwood DA, McCulloch CA. The role of FilGAP-filamin A interactions in mechanoprotection. Mol Biol Cell. 2009;20:1269–1279. 19144823 10.1091/mbc.E08-08-0872 PMC2649276

[45] Razinia Z, Makela T, Ylanne J, Calderwood DA. Filamins in mechanosensing and signaling. Annu Rev Biophys. 2012;41:227–246. 22404683 10.1146/annurev-biophys-050511-102252 PMC5508560

[46] Feng Y, Chen MH, Moskowitz IP, et al Filamin A (FLNA) is required for cell-cell contact in vascular development and cardiac morphogenesis. Proc Natl Acad Sci U S A. 2006;103:19836–19841. 17172441 10.1073/pnas.0609628104 PMC1702530

[47] Carabalona A, Beguin S, Pallesi-Pocachard E, et al A glial origin for periventricular nodular heterotopia caused by impaired expression of Filamin-A. Hum Mol Genet. 2012;21:1004–1017. 22076441 10.1093/hmg/ddr531

[48] Flynn KC, Hellal F, Neukirchen D, et al ADF/cofilin-mediated actin retrograde flow directs neurite formation in the developing brain. Neuron. 2012;76:1091–1107. 23259946 10.1016/j.neuron.2012.09.038

[49] Dent EW, Gertler FB. Cytoskeletal dynamics and transport in growth cone motility and axon guidance. Neuron. 2003;40:209–227. 14556705 10.1016/s0896-6273(03)00633-0

[50] Zheng L, Michelson Y, Freger V, et al Drosophila Ten-m and filamin affect motor neuron growth cone guidance. PLoS One. 2011;6:e22956. 21857973 10.1371/journal.pone.0022956 PMC3152545

[51] Zhang JP, Neal J, Lian GW, Hu JJ, Lu J, Sheen V. Filamin A regulates neuronal migration through brefeldin A-inhibited guanine exchange factor 2-dependent arf1 activation. Journal of Neuroscience. 2013;33:15735–15746. 24089482 10.1523/JNEUROSCI.1939-13.2013 PMC3787497

[52] Zhang J, Neal J, Lian G, Shi B, Ferland RJ, Sheen V. Brefeldin A-inhibited guanine exchange factor 2 regulates filamin A phosphorylation and neuronal migration. J Neurosci. 2012;32:12619–12629. 22956851 10.1523/JNEUROSCI.1063-12.2012 PMC3478955

[53] Sarkisian MR, Bartley CM, Chi H, et al MEKK4 signaling regulates filamin expression and neuronal migration. Neuron. 2006;52:789–801. 17145501 10.1016/j.neuron.2006.10.024 PMC1876745

[54] Zhang LB, Huang TX, Bordey A. Haploinsufficiency is sufficient to increase dendritic patterning and Filamin A levels. Neurosci Lett. 2016;629:15–18. 27345385 10.1016/j.neulet.2016.06.037 PMC4983256

[55] Segura I, Lange C, Knevels E, et al The oxygen sensor PHD2 controls dendritic spines and synapses via modification of Filamin A. Cell Rep. 2016;14:2653–2667. 26972007 10.1016/j.celrep.2016.02.047 PMC4805856

[56] Jobin ML, Siddig S, Koszegi Z, et al Filamin A organizes γ-aminobutyric acid type B receptors at the plasma membrane. Nat Commun. 2023;14:34. 36596803 10.1038/s41467-022-35708-1 PMC9810740

[57] Nakamura F . The role of mechanotransduction in contact inhibition of locomotion and proliferation. Int J Mol Sci. 2024;25:2135. 38396812 10.3390/ijms25042135 PMC10889191

[58] Geiger B, Spatz JP, Bershadsky AD. Environmental sensing through focal adhesions. Nat Rev Mol Cell Bio. 2009;10:21–33. 19197329 10.1038/nrm2593

[59] Evans EA, Calderwood DA. Forces and bond dynamics in cell adhesion. Science. 2007;316:1148–1153. 17525329 10.1126/science.1137592

[60] DuFort CC, Paszek MJ, Weaver VM. Balancing forces: Architectural control of mechanotransduction. Nat Rev Mol Cell Biol. 2011;12:308–319. 21508987 10.1038/nrm3112 PMC3564968

[61] Chighizola M, Dini T, Lenardi C, Milani P, Podesta A, Schulte C. Mechanotransduction in neuronal cell development and functioning. Biophys Rev. 2019;11:701–720. 31617079 10.1007/s12551-019-00587-2 PMC6815321

[62] Zhou J, Kang XM, An HX, Lv Y, Liu X. The function and pathogenic mechanism of filamin A. Gene. 2021;784:145575. 33737122 10.1016/j.gene.2021.145575

[63] Sutherland-Smith AJ . Filamin structure, function and mechanics: Are altered filamin-mediated force responses associated with human disease? Biophys Rev. 2011;3:15–23. 28510233 10.1007/s12551-011-0042-y PMC5425660

[64] Stouffer MA, Golden JA, Francis F. Neuronal migration disorders: Focus on the cytoskeleton and epilepsy. Neurobiol Dis. 2016;92(Pt A):18–45. 26299390 10.1016/j.nbd.2015.08.003 PMC6508100

[65] Subramanian L, Calcagnotto ME, Paredes MF. Cortical malformations: Lessons in human brain development. Front Cell Neurosci. 2019;13:576. 32038172 10.3389/fncel.2019.00576 PMC6993122

[66] Guerrini R, Mei D, Sisodiya S, et al Germline and mosaic mutations of in men with periventricular heterotopia. Neurology. 2004;63:51–56. 15249610 10.1212/01.wnl.0000132818.84827.4d

[67] Yang L, Wu GS, Yin HM, Pan ML, Zhu YF. Periventricular nodular heterotopias is associated with mutation at the locus-a case history and a literature review. Bmc Pediatr. 2023;23:346. 37422633 10.1186/s12887-023-04161-4 PMC10329368

[68] Parrini E, Ramazzotti A, Dobyns WB, et al Periventricular heterotopia: Phenotypic heterogeneity and correlation with Filamin A mutations. Brain. 2006;129(Pt 7):1892–1906. 16684786 10.1093/brain/awl125

[69] Carton RJ, Doyle MG, Kearney H, et al Somatic variants as a cause of drug-resistant epilepsy including mesial temporal lobe epilepsy with hippocampal sclerosis. Epilepsia. 2024;65:1451–1461. 38491957 10.1111/epi.17943 PMC12962753

[70] Li Z, Van Aelst L, Cline HT. Rho GTPases regulate distinct aspects of dendritic arbor growth in Xenopus central neurons in vivo. Nat Neurosci. 2000;3:217–225. 10700252 10.1038/72920

[71] Stankiewicz TR, Linseman DA. Rho family GTPases: Key players in neuronal development, neuronal survival, and neurodegeneration. Front Cell Neurosci. 2014;8:314. 25339865 10.3389/fncel.2014.00314 PMC4187614

[72] Levert S, Pilliod J, Aumont E, et al Direct and indirect effects of filamin A on tau pathology in neuronal cells. Mol Neurobiol. 2023;60:1021–1039. 36399251 10.1007/s12035-022-03121-w PMC9849303

[73] Iffland PH 2nd, Crino PB. Focal cortical dysplasia: Gene mutations, cell signaling, and therapeutic implications. Annu Rev Pathol. 2017;12:547–571. 28135561 10.1146/annurev-pathol-052016-100138

[74] Lord C, Brugha TS, Charman T, et al Autism spectrum disorder. Nat Rev Dis Primers. 2020;6:5. 31949163 10.1038/s41572-019-0138-4 PMC8900942

[75] Gilbert J, Man HY. Fundamental elements in autism: From neurogenesis and neurite growth to synaptic plasticity. Front Cell Neurosci. 2017;11:359. 29209173 10.3389/fncel.2017.00359 PMC5701944

[76] Yang DYJ, Beam D, Pelphrey KA, Abdullahi S, Jou RJ. Cortical morphological markers in children with autism: A structural magnetic resonance imaging study of thickness, area, volume, and gyrification. Mol Autism. 2016;7:11. 26816612 10.1186/s13229-016-0076-x PMC4727390

[77] Pan YH, Wu N, Yuan XB. Toward a better understanding of neuronal migration deficits in autism Spectrum disorders. Front Cell Dev Biol. 2019;7:205. 31620440 10.3389/fcell.2019.00205 PMC6763556

[78] Stoner R, Chow ML, Boyle MP, et al Patches of disorganization in the neocortex of children with autism. N Engl J Med. 2014;370:1209–1219. 24670167 10.1056/NEJMoa1307491 PMC4499461

[79] Reiner O, Karzbrun E, Kshirsagar A, Kaibuchi K. Regulation of neuronal migration, an emerging topic in autism spectrum disorders. J Neurochem. 2016;136:440–456. 26485324 10.1111/jnc.13403

[80] Parikshak NN, Luo R, Zhang A, et al Integrative functional genomic analyses implicate specific molecular pathways and circuits in autism. Cell. 2013;155:1008–1021. 24267887 10.1016/j.cell.2013.10.031 PMC3934107

[81] Caruso A, Ricceri L, Scattoni ML. Ultrasonic vocalizations as a fundamental tool for early and adult behavioral phenotyping of autism Spectrum disorder rodent models. Neurosci Biobehav R. 2020;116:31–43. 10.1016/j.neubiorev.2020.06.011 32544538

[82] Esposito G, Hiroi N, Scattoni ML. Cry, baby, cry: Expression of distress as a biomarker and modulator in autism Spectrum disorder. Int J Neuropsychoph. 2017;20:498–503. 10.1093/ijnp/pyx014 PMC5458334 28204487

[83] Burns LH, Wang HY. Altered filamin A enables amyloid beta-induced tau hyperphosphorylation and neuroinflammation in Alzheimer's disease. Neuroimmunol Neuroinflamm. 2017;4:263–271. 34295950 10.20517/2347-8659.2017.50 PMC8294116

[84] Wang HY, Cecon E, Dam J, Pei Z, Jockers R, Burns LH. Simufilam reverses aberrant receptor interactions of Filamin A in Alzheimer's disease. Int J Mol Sci. 2023;24:13927. 37762230 10.3390/ijms241813927 PMC10531384

[85] Tsujikawa K, Hamanaka K, Riku Y, et al Actin-binding protein filamin-A drives tau aggregation and contributes to progressive supranuclear palsy pathology. Sci Adv. 2022;8:eabm5029. 35613261 10.1126/sciadv.abm5029 PMC9132466

[86] Zada A, Zhao Y, Halim D, et al The long Filamin-A isoform is required for intestinal development and motility: Implications for chronic intestinal pseudo-obstruction. Hum Mol Genet. 2023;32:151–160. 35981053 10.1093/hmg/ddac199 PMC9838097

[87] Gargiulo A, Auricchio R, Barone MV, et al Filamin A is mutated in X-linked chronic idiopathic intestinal pseudo-obstruction with central nervous system involvement. Am J Hum Genet. 2007;80:751–758. 17357080 10.1086/513321 PMC1852717

[88] Kapur RP, Robertson SP, Hannibal MC, et al Diffuse abnormal layering of small intestinal smooth muscle is present in patients with FLNA mutations and x-linked intestinal pseudo-obstruction. Am J Surg Pathol. 2010;34:1528–1543. 20871226 10.1097/PAS.0b013e3181f0ae47

[89] Billon C, Adham S, Poblete NH, et al Cardiovascular and connective tissue disorder features in FLNA-related PVNH patients: Progress towards a refined delineation of this syndrome. Orphanet J Rare Dis. 2021;16:504. 34863227 10.1186/s13023-021-02128-1 PMC8642866

[90] Reinstein E, Frentz S, Morgan T, et al Vascular and connective tissue anomalies associated with X-linked periventricular heterotopia due to mutations in Filamin A. Eur J Hum Genet. 2013;21:494–502. 23032111 10.1038/ejhg.2012.209 PMC3641385

[91] Sasaki E, Byrne AT, Phelan E, Cox DW, Reardon W. A review of filamin A mutations and associated interstitial lung disease. Eur J Pediatr. 2019;178:121–129. 30547349 10.1007/s00431-018-3301-0

[92] de Wit MC, Tiddens HA, de Coo IF, Mancini GM. Lung disease in FLNA mutation: Confirmatory report. Eur J Med Genet. 2011;54:299–300. 21194575 10.1016/j.ejmg.2010.12.009

[93] Deng X, Li S, Qiu Q, et al Where the congenital heart disease meets the pulmonary arterial hypertension, FLNA matters: A case report and literature review. Bmc Pediatr. 2020;20:504. 33143682 10.1186/s12887-020-02393-2 PMC7607646

