# Supplementary material for: Filamin A in focus: unravelling the multifaceted roles of filamin A in neurodevelopment and neurological disorders
Source: Brain. 2025 May 14;148(10):3473–80. doi: 10.1093/brain/awaf180 (PMC12493046; doi:10.1093/brain/awaf180)
Supplement: awaf180_Supplementary_Data [file awaf180_supplementary_data.pdf]

<u>Supplementary Material for:</u> Filamin A (FLNA) in focus: unraveling the multifaceted roles of FLNA in neurodevelopment and neurological disorders

Longbo Zhang

Supplementary Table S1. Interaction partners of filamin

**Binding partner**	**Significance**	**Binding site on filamin**	**Refs**
Androgen receptor	Downregulates androgen receptor in nucleus	Repeats 16-24 after cleavage	[1,2]
ARHGAP24	Rho- and ROCK-regulated GAP for Rac. FLNA-binding is required for cell spreading and stimulates GAP activity	Repeat 23 (A specific)	[3]
AKP13	Co-immunorecipitated with calcium-sensing receptor, RhoA, and Gαq	Unknown	[4]
BRCA-2	Promotes recovery from G2 arrest after DNA damage	Repeats 21-24 in nucleus	[5,6]
Calcitonin receptor	Anchoring and receptor internalization and recycling	Repeats 20-22	[7]
CaR extracellular Ca^2+ receptor	Receptor to actin anchoring	Repeats 14-16	[8]
CAV1	Anchoring caveolae to cytoskeleton	Repeats 22-24	[9]
cvHSP		Repeats 21-24	[10]
CALM	Regulates F-actin binding in vitro	ABD	[11]
CEACAM1	Reduces cell migration	Repeats 23-24	[12]
D2/D3 dopamine receptors	Receptor to actin anchoring	Repeat 19	[13]
ECSCR	Endothelial chemotaxis and tube formation	Repeats 15-16, 19-21	[14]
FILIP1L (Filamin A Interacting Protein 1 Like)	Overexpression of FILIP1L inhibits cell proliferation and migration and increased apoptosis	Unknown	[15]
FIP	Membrane to actin link, signal transduction	Repeats 2-4	[16]
FILIP	Negative regulation of FLNA expression	Repeats 15-18	[17,18]
FURIN	Sorting, compartmentalization, and stabilization	Unknown	[19]
FAP52	Unkown	Repeats 13-16	[20]
FOXC1	Nuclear scaffold	aa 571-866 aa 867-1154 aa 1779-2284	[21]
FcR1 (CD64)	Ligand-sensitive dissociation		[22]
F-actin	Induces orthogonal F-actin networks with unique mechanical and physiological properties	ABD, rod-1	[23]
GPIbα	Transport of GPIbα from endoplasmic reticulum to cell surface	aa 567-571	[24,25]

Glutamate receptor type 7	Receptor to actin anchoring	Repeats 21-22	26
Granzyme B	Participates in granzyme B-mediated apoptosis	Repeats 24	27
HCN1	Receptor to actin anchoring	Repeat 24	28
Integrin	Receptor to actin anchoring	Repeats 19-24	29,30
Insulin receptor	Alters insulin-dependent activation of the mitogen-activated protein kinase pathway	Unknown	18,31
IKAP (ELP1)	Loss-of-function mutations in the IKBKAP gene, which encodes IKAP, cause familial dysautonomia	Unknown	32
Kir2.1	Receptor to actin anchoring; Promotes current density mediated by this specific channel	Repeats 23-24	33,34
Migfilin	Cell adhesion structure to cytoskeleton binding	Repeat 21	35,36
PSN1	Unknow	Carboxyl-terminus	37
PEBP2/CBF	Retains PEBP2 in cytoplasm inhibiting its nuclear activity	Repeats 23-24	38
PAK1	Ruffle formation	Repeat 23	39
Protein kinase Cα	Scaffold for signaling pathway	Repeats 1-3; hinge 2 to repeat 24	40
pro-PrP	FLNA interacts with the GPI anchor peptide signal sequence of pro-PrP that is expressed in some cancer cells. This interaction also promotes cell spreading and migration of melanoma cells	Repeats 10,16-18, 20, 21, 23	41,42
P190RhoGAP	Expression of calpain-insensitive FLNA excludes P190RhoGAP from the lipid raft, thereby increase Rho activity	Unknown	43
P311	Highly expressed in invasive glioma cells and enhances glioma cell migration	Unknown	44
ROCK	Remodeling of cytoskeleton	Repeat 24	45
RALA	Cytoskeleton regulation, filopodia formation	Repeat 24	46
RHO, RAC, CDC 42	Remodeling of cytoskeleton	Repeats 21-24	46,47
RRAS	Enhances integrin activation	Repeat 3	48
SVIL	Overexpression of igFlna8-10, but not 20–22 decreases spreading of Hela cells on fibronectin	Repeats 8-10, 20–22	49
SPHK1	FLNA-dependent kinase activity	Repeats 22-24	50
SHIP-2	Receptor to actin anchoring	Repeats 21-23	18,51
SEK-1	Tumor necrosis factor-α activation	Repeats 21-23	47
SMAD	Anchoring and phosphorylation promotion	Repeats 20-23	52
TRIO	Promotes dorsal ruffling	Repeats 23-24	53
TRAF1, TRAF2	Anchoring and receptor internalization and recycling	Repeats 15-19	54
Tissue factor	Phosphorylation of TF enhances the interaction	Repeats 23-24	18,55
Tc-mip	FLNA is a partner of Tc-mip		18,56
Tyrosine-protein kinase SYK	FLNA is required for ITAM-mediated receptor signaling in platelet	Repeat 5	57
Vimentin	Expression of igFlna1-8 restores spreading of filamin-deficient Hek-293 cells, vimentin phosphorylation, and the cell surface expression of β1 integrins	Repeats 1-8	58

Abbreviations: aa, amino acid residues

References:

1. Loy CJ, Sim KS, Yong EL. Filamin-A fragment localizes to the nucleus to regulate androgen receptor and coactivator functions. *Proc Natl Acad Sci U S A*. Apr 15 2003;100(8):4562-7. doi:10.1073/pnas.0736237100
2. Ozanne DM, Brady ME, Cook S, Gaughan L, Neal DE, Robson CN. Androgen receptor nuclear translocation is facilitated by the f-actin cross-linking protein filamin. *Mol Endocrinol*. Oct 2000;14(10):1618-26. doi:10.1210/mend.14.10.0541
3. Nakamura F, Heikkinen O, Pentikäinen OT*, et al*. Molecular basis of filamin A-FilGAP interaction and its impairment in congenital disorders associated with filamin A mutations. *PLoS One*. 2009;4(3):e4928. doi:10.1371/journal.pone.0004928
4. Pi M, Spurney RF, Tu Q, Hinson T, Quarles LD. Calcium-sensing receptor activation of rho involves filamin and rho-guanine nucleotide exchange factor. *Endocrinology*. Oct 2002;143(10):3830-8. doi:10.1210/en.2002-220240
5. Yuan Y, Shen Z. Interaction with BRCA2 suggests a role for filamin-1 (hsFLNa) in DNA damage response. *J Biol Chem*. Dec 21 2001;276(51):48318-24. doi:10.1074/jbc.M102557200
6. Meng X, Yuan Y, Maestas A, Shen Z. Recovery from DNA damage-induced G2 arrest requires actin-binding protein filamin-A/actin-binding protein 280. *J Biol Chem*. Feb 13 2004;279(7):6098-105. doi:10.1074/jbc.M306794200
7. Seck T, Baron R, Horne WC. Binding of filamin to the C-terminal tail of the calcitonin receptor controls recycling. *J Biol Chem*. Mar 21 2003;278(12):10408-16. doi:10.1074/jbc.M209655200
8. Awata H, Huang C, Handlogten ME, Miller RT. Interaction of the calcium-sensing receptor and filamin, a potential scaffolding protein. *J Biol Chem*. Sep 14 2001;276(37):34871-9. doi:10.1074/jbc.M100775200
9. Stahlhut M, van Deurs B. Identification of filamin as a novel ligand for caveolin-1: evidence for the organization of caveolin-1-associated membrane domains by the actin cytoskeleton. *Mol Biol Cell*. Jan 2000;11(1):325-37. doi:10.1091/mbc.11.1.325
10. Krief S, Faivre JF, Robert P*, et al*. Identification and characterization of cvHsp. A novel human small stress protein selectively expressed in cardiovascular and insulin-sensitive tissues. *J Biol Chem*. Dec 17 1999;274(51):36592-600. doi:10.1074/jbc.274.51.36592
11. Nakamura F, Hartwig JH, Stossel TP, Szymanski PT. Ca2+ and calmodulin regulate the binding of filamin A to actin filaments. *J Biol Chem*. Sep 16 2005;280(37):32426-33. doi:10.1074/jbc.M502203200
12. Klaile E, Müller MM, Kannicht C, Singer BB, Lucka L. CEACAM1 functionally interacts with filamin A and exerts a dual role in the regulation of cell migration. *J Cell Sci*. Dec 1 2005;118(Pt 23):5513-24. doi:10.1242/jcs.02660
13. Lin R, Karpa K, Kabbani N, Goldman-Rakic P, Levenson R. Dopamine D2 and D3 receptors are linked to the actin cytoskeleton via interaction with filamin A. *Proc Natl Acad Sci U S A*. Apr 24 2001;98(9):5258-63. doi:10.1073/pnas.011538198
14. Armstrong LJ, Heath VL, Sanderson S*, et al*. ECSM2, an endothelial specific filamin a binding protein that mediates chemotaxis. *Arterioscler Thromb Vasc Biol*. Sep 2008;28(9):1640-6. doi:10.1161/atvbaha.108.162511
15. Kwon M, Hanna E, Lorang D*, et al*. Functional characterization of filamin a interacting protein 1-like, a novel candidate for antivascular cancer therapy. *Cancer Res*. Sep 15 2008;68(18):7332-41. doi:10.1158/0008-5472.Can-08-1087
16. Knuth M, Khaire N, Kuspa A, Lu SJ, Schleicher M, Noegel AA. A novel partner for Dictyostelium filamin is an alpha-helical developmentally regulated protein. *J Cell Sci*. Oct 1 2004;117(Pt 21):5013-22. doi:10.1242/jcs.01366

17. Nagano T, Morikubo S, Sato M. Filamin A and FILIP (Filamin A-Interacting Protein) regulate cell polarity and motility in neocortical subventricular and intermediate zones during radial migration. *J Neurosci*. Oct 27 2004;24(43):9648-57. doi:10.1523/JNEUROSCI.2363-04.2004
18. Feng Y, Walsh CA. The many faces of filamin: a versatile molecular scaffold for cell motility and signalling. *Nat Cell Biol*. Nov 2004;6(11):1034-8. doi:10.1038/ncb1104-1034
19. Liu G, Thomas L, Warren RA*, et al*. Cytoskeletal protein ABP-280 directs the intracellular trafficking of furin and modulates proprotein processing in the endocytic pathway. *J Cell Biol*. Dec 29 1997;139(7):1719-33. doi:10.1083/jcb.139.7.1719
20. Nikki M, Merilainen J, Lehto VP. FAP52 regulates actin organization via binding to filamin. *J Biol Chem*. Mar 29 2002;277(13):11432-40. doi:10.1074/jbc.M111753200
21. Berry FB, O'Neill MA, Coca-Prados M, Walter MA. FOXC1 transcriptional regulatory activity is impaired by PBX1 in a filamin A-mediated manner. *Mol Cell Biol*. Feb 2005;25(4):1415-24. doi:10.1128/MCB.25.4.1415-1424.2005
22. Ohta Y, Stossel TP, Hartwig JH. Ligand-sensitive binding of actin-binding protein to immunoglobulin G Fc receptor I (Fc gamma RI). *Cell*. Oct 18 1991;67(2):275-82. doi:10.1016/0092-8674(91)90179-3
23. Nakamura F, Osborn TM, Hartemink CA, Hartwig JH, Stossel TP. Structural basis of filamin A functions. *J Cell Biol*. Dec 3 2007;179(5):1011-25. doi:10.1083/jcb.200707073
24. Nakamura F, Pudas R, Heikkinen O*, et al*. The structure of the GPIb-filamin A complex. *Blood*. Mar 1 2006;107(5):1925-32. doi:10.1182/blood-2005-10-3964
25. Cranmer SL, Pikovski I, Mangin P*, et al*. Identification of a unique filamin A binding region within the cytoplasmic domain of glycoprotein Ibalpha. *Biochem J*. May 1 2005;387(Pt 3):849-58. doi:10.1042/BJ20041836
26. Enz R. The actin-binding protein Filamin-A interacts with the metabotropic glutamate receptor type 7. *FEBS Lett*. Mar 13 2002;514(2-3):184-8. doi:10.1016/s0014-5793(02)02361-x
27. Browne KA, Johnstone RW, Jans DA, Trapani JA. Filamin (280-kDa actin-binding protein) is a caspase substrate and is also cleaved directly by the cytotoxic T lymphocyte protease granzyme B during apoptosis. *J Biol Chem*. Dec 15 2000;275(50):39262-6. doi:10.1074/jbc.C000622200
28. Gravante B, Barbuti A, Milanesi R, Zappi I, Viscomi C, DiFrancesco D. Interaction of the pacemaker channel HCN1 with filamin A. *J Biol Chem*. Oct 15 2004;279(42):43847-53. doi:10.1074/jbc.M401598200
29. Kiema T, Lad Y, Jiang P*, et al*. The molecular basis of filamin binding to integrins and competition with talin. *Mol Cell*. Feb 3 2006;21(3):337-47. doi:10.1016/j.molcel.2006.01.011
30. Travis MA, van der Flier A, Kammerer RA, Mould AP, Sonnenberg A, Humphries MJ. Interaction of filamin A with the integrin beta 7 cytoplasmic domain: role of alternative splicing and phosphorylation. *FEBS Lett*. Jul 2 2004;569(1-3):185-90. doi:10.1016/j.febslet.2004.04.099
31. He HJ, Kole S, Kwon YK, Crow MT, Bernier M. Interaction of filamin A with the insulin receptor alters insulin-dependent activation of the mitogen-activated protein kinase pathway. *J Biol Chem*. Jul 18 2003;278(29):27096-104. doi:10.1074/jbc.M301003200
32. Johansen LD, Naumanen T, Knudsen A*, et al*. IKAP localizes to membrane ruffles with filamin A and regulates actin cytoskeleton organization and cell migration. *J Cell Sci*. Mar 15 2008;121(Pt 6):854-64. doi:10.1242/jcs.013722
33. Sampson LJ, Leyland ML, Dart C. Direct interaction between the actin-binding protein filamin-A and the inwardly rectifying potassium channel, Kir2.1. *J Biol Chem*. Oct 24 2003;278(43):41988-97. doi:10.1074/jbc.M307479200
34. Petrecca K, Miller DM, Shrier A. Localization and enhanced current density of the Kv4.2 potassium channel by interaction with the actin-binding protein filamin. *J Neurosci*. Dec 1 2000;20(23):8736-44. doi:10.1523/JNEUROSCI.20-23-08736.2000

35. Tu Y, Wu S, Shi X, Chen K, Wu C. Migfilin and Mig-2 link focal adhesions to filamin and the actin cytoskeleton and function in cell shape modulation. *Cell*. Apr 4 2003;113(1):37-47. doi:10.1016/s0092-8674(03)00163-6

36. Wu C. Migfilin and its binding partners: from cell biology to human diseases. *J Cell Sci*. Feb 15 2005;118(Pt 4):659-64. doi:10.1242/jcs.01639

37. Guo Y, Zhang SX, Sokol N, Cooley L, Boulianne GL. Physical and genetic interaction of filamin with presenilin in Drosophila. *J Cell Sci*. Oct 2000;113 Pt 19:3499-508. doi:10.1242/jcs.113.19.3499

38. Yoshida N, Ogata T, Tanabe K*, et al*. Filamin A-bound PEBP2beta/CBFbeta is retained in the cytoplasm and prevented from functioning as a partner of the Runx1 transcription factor. *Mol Cell Biol*. Feb 2005;25(3):1003-12. doi:10.1128/MCB.25.3.1003-1012.2005

39. Vadlamudi RK, Li F, Adam L*, et al*. Filamin is essential in actin cytoskeletal assembly mediated by p21-activated kinase 1. *Nat Cell Biol*. Sep 2002;4(9):681-90. doi:10.1038/ncb838

40. Tigges U, Koch B, Wissing J, Jockusch BM, Ziegler WH. The F-actin cross-linking and focal adhesion protein filamin A is a ligand and in vivo substrate for protein kinase C alpha. *J Biol Chem*. Jun 27 2003;278(26):23561-9. doi:10.1074/jbc.M302302200

41. Li C, Yu S, Nakamura F*, et al*. Pro-prion binds filamin A, facilitating its interaction with integrin beta1, and contributes to melanomagenesis. *J Biol Chem*. Sep 24 2010;285(39):30328-39. doi:10.1074/jbc.M110.147413

42. Li C, Yu S, Nakamura F*, et al*. Binding of pro-prion to filamin A disrupts cytoskeleton and correlates with poor prognosis in pancreatic cancer. *J Clin Invest*. Sep 2009;119(9):2725-36. doi:10.1172/jci39542

43. Mammoto A, Huang S, Ingber DE. Filamin links cell shape and cytoskeletal structure to Rho regulation by controlling accumulation of p190RhoGAP in lipid rafts. *J Cell Sci*. Feb 1 2007;120(Pt 3):456-67. doi:10.1242/jcs.03353

44. McDonough WS, Tran NL, Berens ME. Regulation of glioma cell migration by serine-phosphorylated P311. *Neoplasia*. Sep 2005;7(9):862-72. doi:10.1593/neo.05190

45. Ueda K, Ohta Y, Hosoya H. The carboxy-terminal pleckstrin homology domain of ROCK interacts with filamin-A. *Biochem Biophys Res Commun*. Feb 21 2003;301(4):886-90. doi:10.1016/s0006-291x(03)00048-2

46. Ohta Y, Suzuki N, Nakamura S, Hartwig JH, Stossel TP. The small GTPase RalA targets filamin to induce filopodia. *Proc Natl Acad Sci U S A*. Mar 2 1999;96(5):2122-8. doi:10.1073/pnas.96.5.2122

47. Marti A, Luo Z, Cunningham C*, et al*. Actin-binding protein-280 binds the stress-activated protein kinase (SAPK) activator SEK-1 and is required for tumor necrosis factor-alpha activation of SAPK in melanoma cells. *J Biol Chem*. Jan 31 1997;272(5):2620-8. doi:10.1074/jbc.272.5.2620

48. Gawecka JE, Griffiths GS, Ek-Rylander B, Ramos JW, Matter ML. R-Ras regulates migration through an interaction with filamin A in melanoma cells. *PLoS One*. Jun 23 2010;5(6):e11269. doi:10.1371/journal.pone.0011269

49. Smith TC, Fang Z, Luna EJ. Novel interactors and a role for supervillin in early cytokinesis. *Cytoskeleton (Hoboken)*. Jun 2010;67(6):346-64. doi:10.1002/cm.20449

50. Maceyka M, Alvarez SE, Milstien S, Spiegel S. Filamin A links sphingosine kinase 1 and sphingosine-1-phosphate receptor 1 at lamellipodia to orchestrate cell migration. *Mol Cell Biol*. Sep 2008;28(18):5687-97. doi:10.1128/mcb.00465-08

51. Dyson JM, Munday AD, Kong AM*, et al*. SHIP-2 forms a tetrameric complex with filamin, actin, and GPIb-IX-V: localization of SHIP-2 to the activated platelet actin cytoskeleton. *Blood*. Aug 1 2003;102(3):940-8. doi:10.1182/blood-2002-09-2897

52. Sasaki A, Masuda Y, Ohta Y, Ikeda K, Watanabe K. Filamin associates with Smads and regulates transforming growth factor-beta signaling. *J Biol Chem*. May 25 2001;276(21):17871-7. doi:10.1074/jbc.M008422200

53. Bellanger JM, Astier C, Sardet C, Ohta Y, Stossel TP, Debant A. The Rac1- and RhoG-specific GEF domain of Trio targets filamin to remodel cytoskeletal actin. *Nat Cell Biol*. Dec 2000;2(12):888-92. doi:10.1038/35046533
54. Arron JR, Pewzner-Jung Y, Walsh MC, Kobayashi T, Choi Y. Regulation of the subcellular localization of tumor necrosis factor receptor-associated factor (TRAF)2 by TRAF1 reveals mechanisms of TRAF2 signaling. *J Exp Med*. Oct 7 2002;196(7):923-34. doi:10.1084/jem.20020774
55. Ott I, Fischer EG, Miyagi Y, Mueller BM, Ruf W. A role for tissue factor in cell adhesion and migration mediated by interaction with actin-binding protein 280. *J Cell Biol*. Mar 9 1998;140(5):1241-53. doi:10.1083/jcb.140.5.1241
56. Grimbert P, Valanciute A, Audard V, Lang P, Guellaën G, Sahali D. The Filamin-A is a partner of Tc-mip, a new adapter protein involved in c-maf-dependent Th2 signaling pathway. *Mol Immunol*. Mar 2004;40(17):1257-61. doi:10.1016/j.molimm.2003.11.035
57. Falet H, Pollitt AY, Begonja AJ*, et al*. A novel interaction between FlnA and Syk regulates platelet ITAM-mediated receptor signaling and function. *J Exp Med*. Aug 30 2010;207(9):1967-79. doi:10.1084/jem.20100222
58. Kim H, Nakamura F, Lee W, Hong C, Pérez-Sala D, McCulloch CA. Regulation of cell adhesion to collagen via beta1 integrins is dependent on interactions of filamin A with vimentin and protein kinase C epsilon. *Exp Cell Res*. Jul 1 2010;316(11):1829-44. doi:10.1016/j.yexcr.2010.02.007
